# Acute Myocarditis Secondary to Paediatric Inflammatory Multisystem Syndrome Temporally Associated With COVID-19 Infection

**DOI:** 10.7759/cureus.22420

**Published:** 2022-02-20

**Authors:** Chaitya Desai, Sunil Aggarwal, Ahsan A Khan

**Affiliations:** 1 Urology, University Hospitals of North Midlands NHS Trust, Stoke-on-Trent, GBR; 2 Internal Medicine, University Hospitals Birmingham NHS Foundation Trust, Birmingham, GBR; 3 Cardiology, Sandwell and West Birmingham NHS Trust, Birmingham, GBR

**Keywords:** acute, pims-ts, paediatric clinical cardiology, covid-19, myocarditis

## Abstract

A 17-year-old female, who was previously fit and well with no preexisting health conditions, presented with a four-day history of worsening shortness of breath and diarrhoea. She had recent close contact with a relative diagnosed with COVID-19. On clinical examination, she was drowsy, hypotensive, tachycardic, tachypnoeic, and pyrexial. Her blood tests showed elevated inflammatory markers and lymphopenia. She underwent a transthoracic echocardiogram, which confirmed a severely impaired left ventricular (LV) systolic function with an ejection fraction of 35%. An initial impression of acute viral myocarditis was made. Three separate polymerase chain reaction (PCR) tests for SARS-CoV-2 RNA were performed, but they all returned negative. The patient was not responding to initial therapy. Therefore, the regional paediatrics hospital was consulted, and a diagnosis of paediatric inflammatory multisystem syndrome temporally associated with COVID-19 (PIMS-TS) was made, based on similar regional presentations. The patient was administered IV immunoglobulin therapy, to which she responded very well. Following a five-day hospital stay, the patient was discharged home as medically stable. A repeat transthoracic echocardiogram (TTE) showed recovery of the LV systolic function to 62%. Few cases have been reported on myocardial involvement in young patients with PIMS-TS. This case report highlights the initial presentation, medical care, and clinical course of this patient.

## Introduction

The SARS-CoV-2 is responsible for causing the COVID-19, which was declared a global pandemic by the WHO on 11 March 2020 [1]. In the United Kingdom (UK), Birmingham emerged as having the highest concentration of COVID-19 cases outside of London [2]. Several cardiovascular complications of COVID-19 have been described in the adult population, and acute myocarditis appears to be relatively frequent [3]. However, younger patients may be overlooked for cardiovascular complications with COVID-19 infection, as they may present with atypical symptoms.

On April 27, 2020, the Paediatric Intensive Care Society produced a paper highlighting a growing concern for an increase in paediatric inflammatory multisystem syndrome temporally associated with COVID-19 infection (PIMS-TS) [4]. We report the initial presentation, medical care, and clinical course of a 17-year-old girl who presented with acute myocarditis secondary to PIMS-TS. This report is essential as it highlights an approach to ensuring early diagnosis and appropriate management for this vulnerable patient group.

## Case presentation

History and examination findings

A 17-year-old female of Afro-Caribbean ethnicity presented to the ED at our district general hospital with no past medical history of note or cardiovascular risk factors. She reported contact with her grandmother on May 1, 2020, who had been confirmed as COVID-19 positive on April 25, 2020. Around this time, our patient reported anosmia and ageusia, which subsequently resolved. Subsequently, the patient presented to ED on May 6, 2020, with a 4-day history of shortness of breath on minimal exertion. She also reported fevers, profuse diarrhoea, photophobia, and neck stiffness for the preceding two days. The patient had no recent travel history and reported no possibility of pregnancy. She was unvaccinated against COVID-19 infection as she presented prior to the development of any global vaccinations.

Initial observations done in ED found the patient to be tachycardic (142 beats per minute), tachypnoeic (25 breaths per minute), hypotensive (88/65 mmHg), and pyrexial (38.9 degrees celsius). Her oxygen saturations were 98% on room air. Venous blood gas showed bicarbonate of 20.6 and a lactate of 3.3 but a normal acid-base profile, indicating a metabolic acidosis with full respiratory compensation. On examination, the patient was drowsy but responsive to voice. She demonstrated no objective photophobia, true neck stiffness, or focal neurology.

Investigations

**Figure 1:**
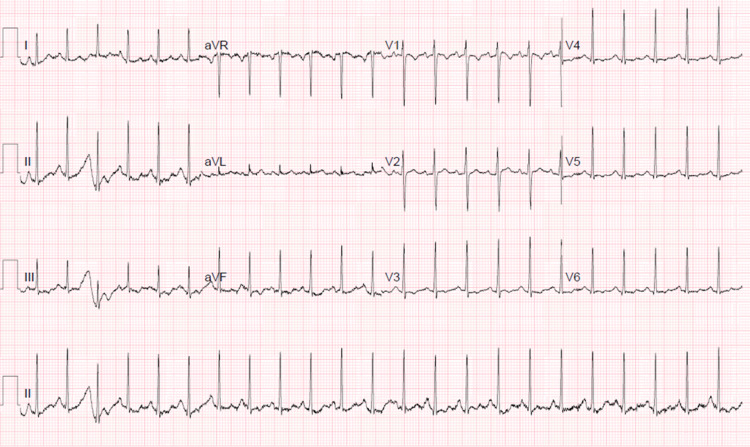
ECG recorded at admission.

The ECG recorded at admission showed sinus tachycardia (Figure 1). Subsequent ECGs recorded during admission remained stable with no evidence of ventricular hypertrophy or ischaemic changes.

**Figure 2:**
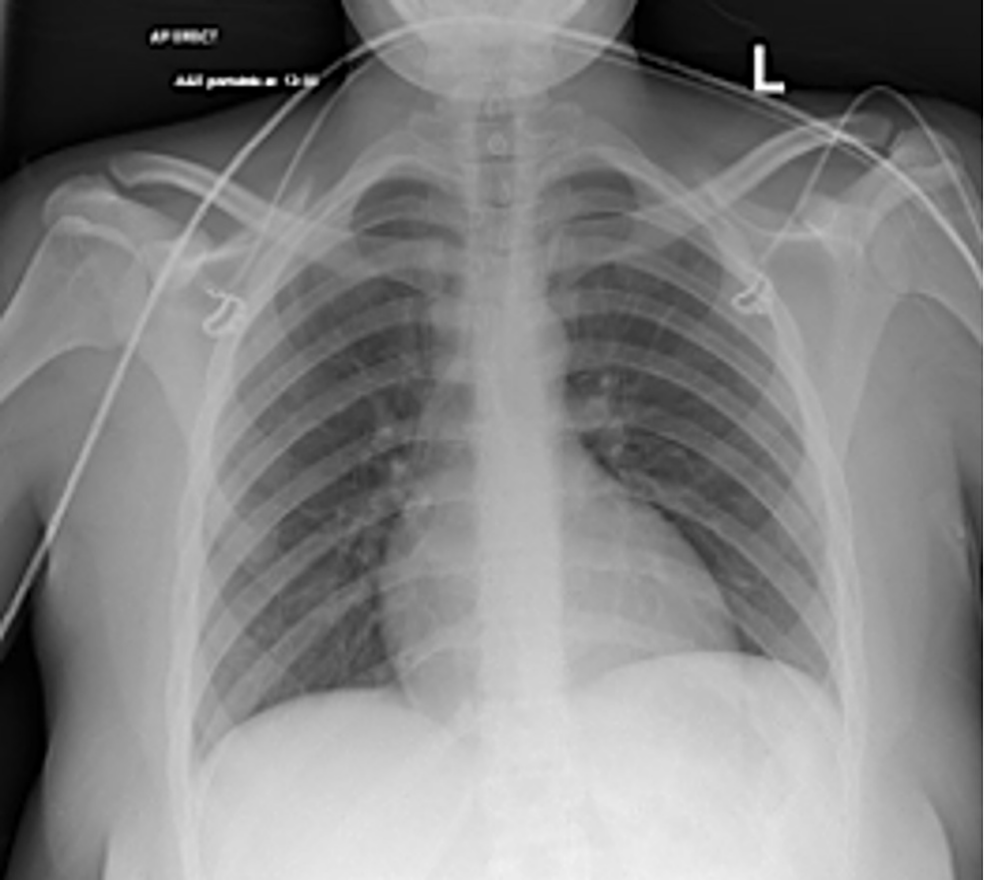
On admission, plain film anteroposterior erect chest radiograph.

A portable chest X-ray was also requested in the ED, and it showed normal lungs with no pathology (Figure 2).

**Figure 3:**
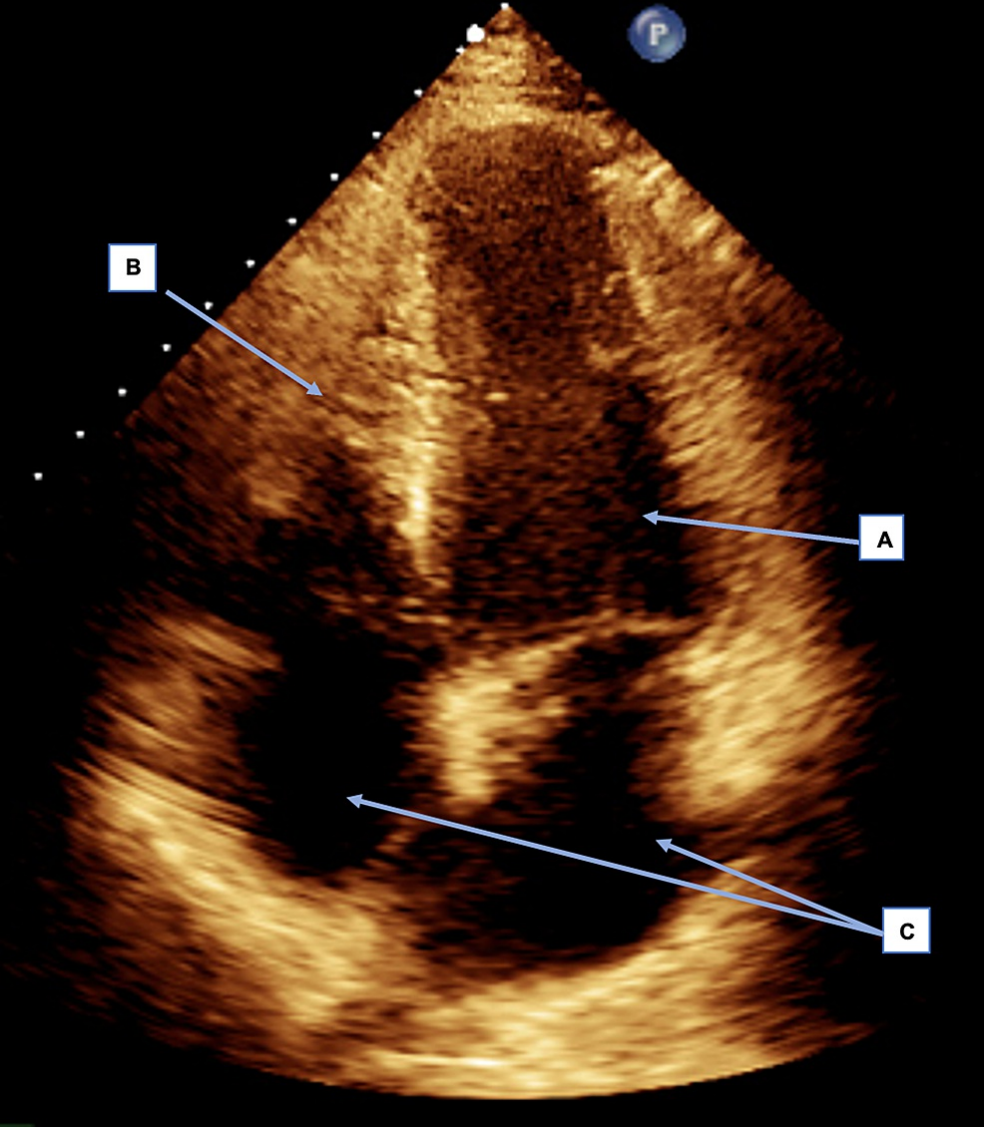
Pre-treatment TTE: Apical 4 chamber (A4C) view in systole showing reduced LV systolic function. A: Left ventricle visually appears dilated in this still image suggestive of impaired function in systole; B: The right ventricle visually appears to be contracting maximally in this still image, suggestive of normal function in systole; C: Both atria appear to be normal in size in this still image. TTE: Transthoracic echocardiogram; LV: Left ventricular.

**Figure 4:**
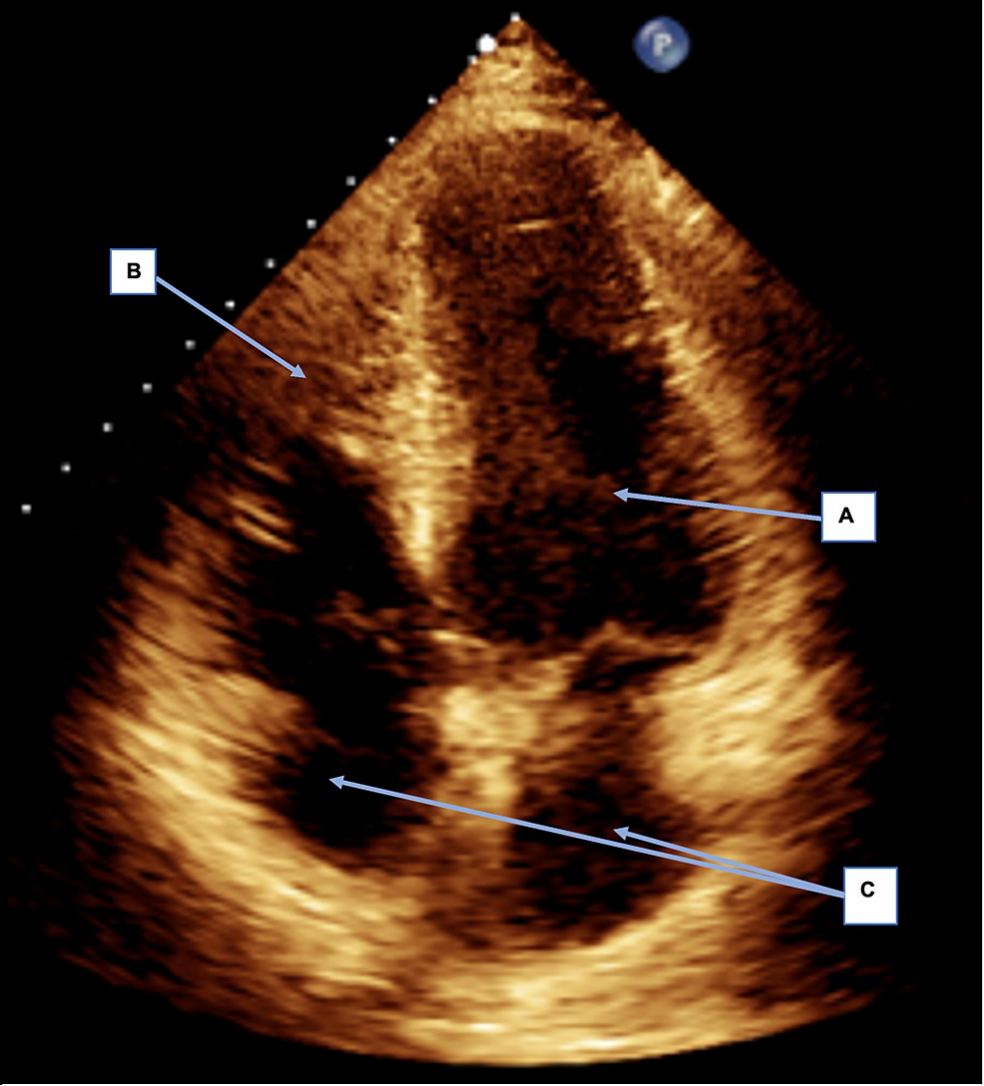
Pre-treatment TTE: Apical 4 chamber (A4C) view in diastole, showing reduced LV systolic function. A: Left ventricle visually appears dilated in this still image, suggestive of impaired function in diastole; B: The right ventricle visually appears to be non-dilated in this still image, suggestive of normal function in diastole; C: Both atria appear to be normal in size in this still image. TTE: Transthoracic echocardiogram; LV: Left ventricular.

Blood tests revealed lymphopenia (0.56 x 109/L) and neutrophilia (10.5 x 109/L), with a marked rise in C-reactive protein (409 mg/L). International normalised ratio and D-dimer were elevated at 1.53 and 1.92 ugFEU/ml, respectively. The cardiac troponin was elevated at 340 ng/L, which reduced to 252 ng/L six hours later. Alanine aminotransferase was elevated at 59 U/L. Blood cultures obtained on admission were negative, as was polymerase chain reaction (PCR) testing for meningococcal and pneumococcal disease. Additionally, a viral screen for Epstein-Barr virus (EBV), Cytomegalovirus (CMV), and Herpes simplex virus (HSV) were all negative. The acute medical registrar performed a point-of-care ultrasound scan, and it showed mild right ventricular dilatation with no valvular abnormalities. A computed tomography pulmonary angiography (CTPA) scan to assess for pulmonary embolism as a possible cause for the right heart strain was requested on admission to the acute medical unit (AMU). The CTPA was negative for any emboli and showed no 'ground-glass' opacification. In view of the recent contact with a COVID-19 confirmed positive case and the patient's prior symptomatic presentation of anosmia and ageusia, there was a high clinical suspicion of COVID-19 infection. Therefore, an urgent nasopharyngeal PCR swab was performed to test for SARS-CoV-2 RNA. Interestingly, the PCR result was negative for the virus. Due to the clinical presentation, the patient was discussed with the local cardiology team. They advised a formal echocardiogram (ECHO) to be conducted to investigate suspected acute viral myocarditis. The ECHO showed a normal-sized left ventricular (LV) function with at least moderate LV systolic dysfunction due to regional wall motion abnormality (RWMA). The LV ejection fraction was estimated at 35% (severely impaired). The right ventricle had no dysfunction noted, and there was no significant valve disease. Z scores for LV End-Diastolic Diameter and LV End-Systolic Diameter were -8.40 and -6.48, respectively. The images of the transthoracic echocardiogram (TTE) performed pre-treatment are shown in Figures 3-4.

Treatment

**Figure 5:**
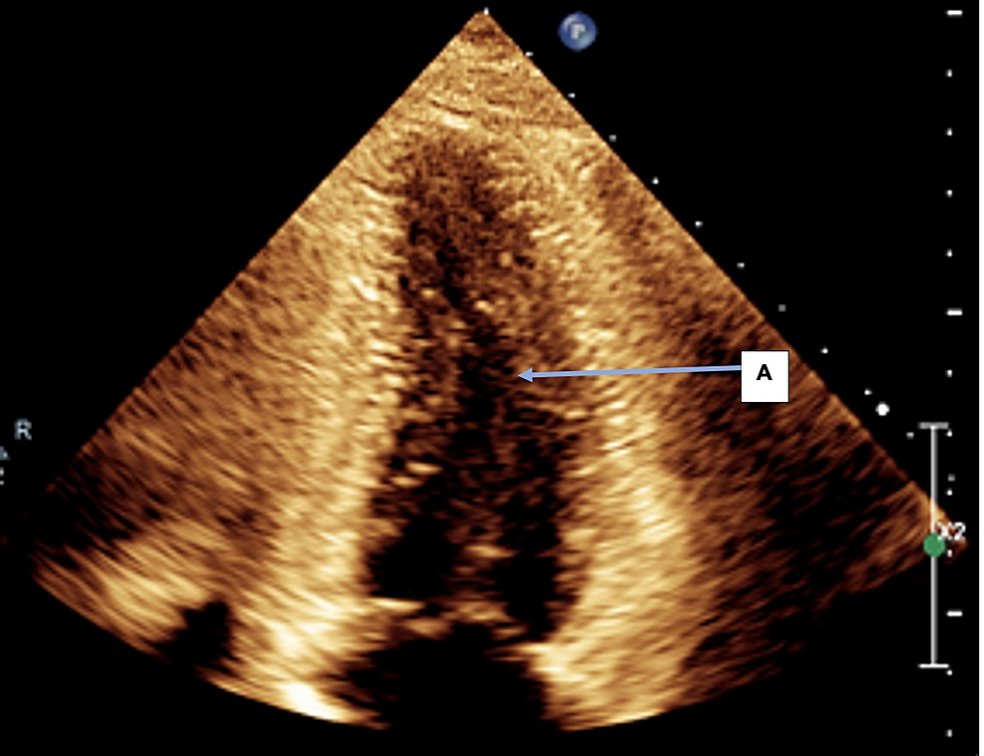
Post-treatment TTE: Apical 4 chamber (A4C) view in systole showing preserved LV systolic function. A: The left ventricle appears to be contracting well in this still image. TTE: Transthoracic echocardiogram; LV: Left ventricular.

**Figure 6:**
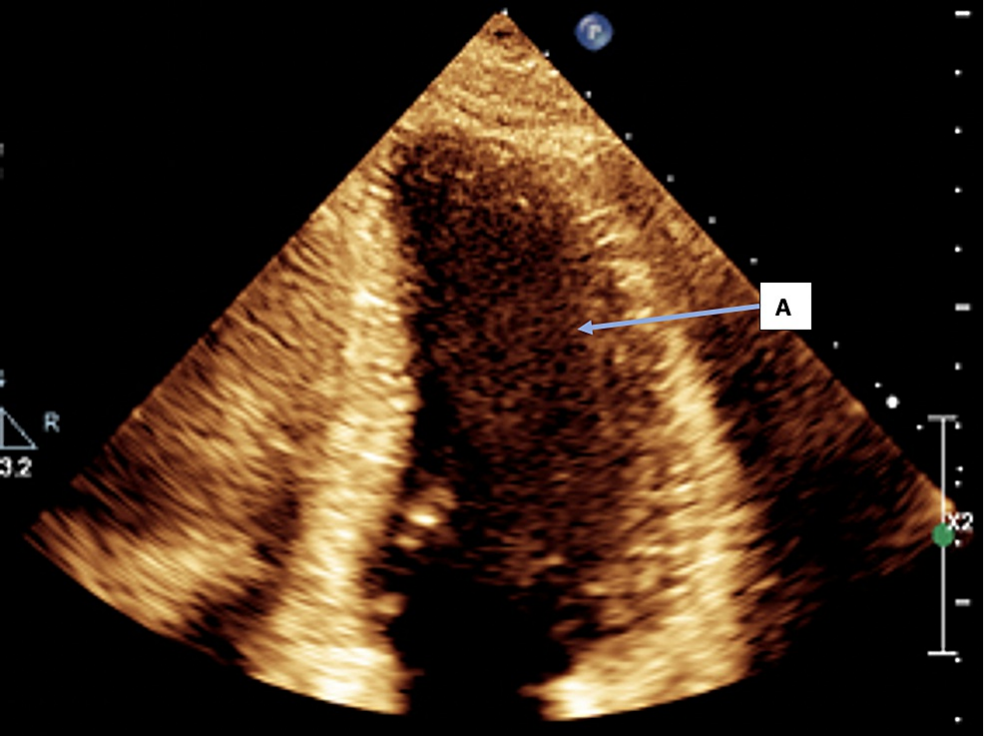
Post-treatment TTE: Apical 4 chamber (A4C) view in diastole showing preserved LV systolic function. A: The left ventricle appears of normal size in diastole in this still image. TTE: Transthoracic echocardiogram; LV: Left ventricular.

Due to the complexity of the case and lack of clarity around a unifying diagnosis, the patient was discussed at a virtual multi-disciplinary team (MDT) meeting. The MDT included members of the parent medical team, cardiology, and specialist paediatrics based at a nearby tertiary centre. The paediatrics team reviewed the case and agreed that the patient fit the phenotype of PIMS-TS despite the negative COVID-19 swab. Subsequently, the patient was commenced on IV immunoglobulin (IVIG) at 2 grams per kilogram (g/kg) over 18 hours and low-dose aspirin 75 mg once daily. She was not considered for angiotensin-converting enzyme inhibitor therapy in view of the acute phase of the syndrome. The haemodynamics of the patient remained stable without indication for inotropic support. She remained afebrile, and the biological inflammatory syndrome gradually decreased. Post-treatment TTE demonstrated recovery of LV systolic function (Figures 5-6). No ventricular arrhythmias were observed during her hospitalisation. High-sensitivity troponin I peaked at 2641 ng/L on day 3.

Outcome and follow-up

The patient was eventually discharged from the hospital on day 8 of her admission after making a good recovery. The case was discussed with the paediatric team, and it was decided that no further follow-up of the patient was required.

## Discussion

We describe a case of a previously healthy adolescent female presenting with haemodynamic instability on a background of exertional dyspnoea, atypical GI symptoms, and drowsiness. Her initial investigations were indicative of myocarditis. Common microbial causes of this presentation were primarily excluded, and after consultation with a specialist paediatric unit, a diagnosis of suspected PIMS-TS was eventually made.

We used the PIMS-TS criteria for this case report as defined by the Royal College of Paediatrics and Child Health published on May 1, 2020 [5]. The definition of PIMS-TS is broad and includes a spectrum of clinical and biochemical features [5]. The main features include persistent fever, inflammation, and evidence of single or multiorgan dysfunction [5]. Crucially, it is stated in the case definition that PCR for SARS-CoV-2 may be positive or negative (as in our case) [5]. Therefore, it is also imperative to exclude other causes of microbial infection which may result in this presentation [5]. In addition, another definition of PIMS-TS has subclassified the condition into 'Kawasaki-disease-like' and 'nonspecific' [6]. The 'nonspecific' group includes patients presenting with symptoms that do not meet the criteria for Kawasaki disease as defined in the American Heart Association (AHA) criteria [7]. Our patient would likely come under the 'nonspecific' group category owing to the presence of atypical GI symptoms and neurological involvement [6]. As of May 2020, approximately 230 suspected cases of PIMS-TS were recorded in Europe, including two fatalities [8].

The main sequelae of PIMS-TS as described in this case was acute myocarditis. We have found other observational studies that reported cardiac involvement in PIMS-TS [9-11]. One case series looked at cases of PIMS-TS from a single UK hospital and found that 80% of the patient cohort had some degree of impaired LV ejection fraction [9]. All patients demonstrated raised cardiac markers indicating some form of myocarditis [9]. Furthermore, a case series from Paris described myocarditis occurring in 70% of its patient cohort [10], while the Bergamo series identified cardiac involvement in 60% [11]. This data shows that myocarditis, amongst other cardiac abnormalities, represents a key component of presenting PIMS-TS in young patients. Early use of echocardiography to assess cardiac function and specialist cardiology review should therefore be performed whenever a diagnosis of PIMS-TS is suspected. This will lead to early identification of possible myocarditis allowing for more timely intervention and improved patient outcomes. What remains unclear from this data is how many patients with PIMS-TS present with myocarditis as the predominant clinical feature. Further research examining this specific relationship would therefore be warranted.

When assessing this case in May 2020, there was no available consensus or guideline available for the workup and management of PIMS-TS. Recently, however, a national consensus for managing this condition based on the opinions of leading clinicians across the UK has been published [6]. For both 'Kawasaki-disease-like' and 'nonspecific' groups, the consensus report advises using IVIG at a dose of 2 g/kg as first-line therapy [6]. A second dose of IVIG can be considered if there is an inadequate or partial response to the first. In our case, the patient responded fully to a single dose of IVIG, leading to clinical and echocardiographic improvement (ejection fraction improved from 35% to 62%). IV Methylprednisolone can be considered a second-line treatment option but was not required in our case. Although our patient also received low-dose aspirin, this has not been recommended in the recent expert consensus [6]. However, the consensus group agreed that early MDT involvement, ideally within 24 h of diagnosis, is essential for management. In our case study, MDT discussions involving key members of the paediatric and cardiology teams were sought early. These discussions enabled a unifying diagnosis to be reached and ensured timely interventions were made. Ultimately, this resulted in an overall positive clinical outcome for this patient.

With regard to the underlying pathophysiology of acute myocarditis in PIMS-TS, there remains much uncertainty. It has been postulated that there may be a similar inflammatory response to that seen in Kawasaki disease. However, further research is needed to characterise this relationship in more detail [12]. Additionally, short-term outcomes with PIMS-TS have been shown to be favourable in patients receiving prompt treatment. However, longer-term outcomes remain unclear due to a lack of sufficient longitudinal data. Therefore, prospective observational cohort studies on individuals with PIMS-TS would be of significant benefit.

## Conclusions

There are several important learning points from this case report. First, children with PIMS-TS may present with atypical symptoms such as diarrhoea or drowsiness. Second, myocarditis may be the only presenting complication of PIMS-TS as in this case. Third, PIMS-TS can be diagnosed despite a negative COVID-19 PCR test as long as clinical features are in keeping with the disease and other microbial causes have been excluded. Early identification of myocardial injury is vital: check cardiac markers, perform an echocardiogram, and seek specialist advice early. Furthermore, favourable clinical outcomes can be achieved with IVIG, and prompt treatment can improve symptoms and cardiac function. Finally, early MDT involvement should be sought. This can guide clinical decision making, diagnosis and treatment initiation.
